# Spatio-temporal trends of mortality in small areas of Southern Spain

**DOI:** 10.1186/1471-2458-10-26

**Published:** 2010-01-20

**Authors:** Ricardo Ocaña-Riola, José María Mayoral-Cortés

**Affiliations:** 1 Escuela Andaluza de Salud Pública, Granada, Spain; 2 Servicio de Epidemiología y Salud Laboral, Consejería de Salud de la Junta de Andalucía, Sevilla, Spain

## Abstract

**Background:**

Most mortality atlases show static maps from count data aggregated over time. This procedure has several methodological problems and serious limitations for decision making in Public Health. The evaluation of health outcomes, including mortality, should be approached from a dynamic time perspective that is specific for each gender and age group. At the moment, researches in Spain do not provide a dynamic image of the population's mortality status from a spatio-temporal point of view. The aim of this paper is to describe the spatial distribution of mortality from all causes in small areas of Andalusia (Southern Spain) and evolution over time from 1981 to 2006.

**Methods:**

A small-area ecological study was devised using the municipality as the unit for analysis. Two spatio-temporal hierarchical Bayesian models were estimated for each age group and gender. One of these was used to estimate the specific mortality rate, together with its time trends, and the other to estimate the specific rate ratio for each municipality compared with Spain as a whole.

**Results:**

More than 97% of the municipalities showed a diminishing or flat mortality trend in all gender and age groups. In 2006, over 95% of municipalities showed male and female mortality specific rates similar or significantly lower than Spanish rates for all age groups below 65. Systematically, municipalities in Western Andalusia showed significant male and female mortality excess from 1981 to 2006 only in age groups over 65.

**Conclusions:**

The study shows a dynamic geographical distribution of mortality, with a different pattern for each year, gender and age group. This information will contribute towards a reflection on the past, present and future of mortality in Andalusia.

## Background

Disease mapping is currently a major research area in Epidemiology. Advances in computer systems, the availability of powerful Geographical Information Systems (GIS) and the implementation of complex mathematical models in specialised software have all encouraged the publication of many ecological small-area studies over the past decade [1]. Here, Hierarchical Bayesian models have played a leading role over the past few years, particularly the Besag-York-Mollié autoregressive conditional model [2].

Since 1970, many atlases have been published around the world describing the geographical distribution of mortality. However, in spite of its popularity, most epidemiological small-area studies show misuse of disease mapping methods. Firstly, nearly all of them describe the geographical distribution of mortality by grouping year on year data into a single period. Some studies have even used periods spanning over 20 years, providing a very static view of mortality that occasionally groups together years when very distinct health plans and related legislation were in force. The use of time periods may also lead to a bias in the estimates for relative risk, such that the excess deaths seen in certain small-areas may only be reflecting a past situation that, due to the aggregated nature of the information, continues to emerge [3].

Secondly, most atlases use age-adjusted rates or standardised mortality rates. However, these indicators are invalid unless all stratum-specific rates of each small-area are a constant multiple of both the specific reference population rates and the specific rates of the other small-areas [4]. In other words, a standardised indicator is not appropriate because the geographical distribution of mortality uses to be different in each stratum [5-7].

As a result, decision-making and health policies based on the interpretation of static maps and aggregate information may be inappropriate, and excess mortality could simply be a consequence of a misuse of statistical methods.

Health phenomena are dynamic and most developed countries have experienced radical, rapid changes over the past few decades. Health determinants, healthcare technology and healthcare resources change over time and, in turn, also have repercussions for the population's welfare [8]. So, the evaluation of health outcomes, including mortality, should be approached from a dynamic time perspective that is specific for each gender and age group. This is the best way to describe trends in health indicators, to evaluate the repercussions of past health policies and to ascertain the current health status of the population so that future improvements may be undertaken [9].

At the moment, there is not any research in Spain that provides both a historical and a dynamic image of the population's health status from a spatio-temporal point of view.

For these reasons, the aim of this paper is to describe the spatial distribution of mortality from all causes in of Andalusia (Southern Spain) and evolution over time from 1981 to 2006.

## Methods

### Design

A small-area ecological study was devised using the municipality as the unit for analysis. The study was carried out in Andalusia (Southern Spain), comprising 770 municipalities with a population of 8,202,220 inhabitants (4,071,500 men and 4,130,720 women). It is the Autonomous Region in Spain with the largest territorial surface and highest population figures. In 2007, Andalusia recorded 18% of the overall national population as resident, and 17% of all deaths occurring in Spain [10].

### Variables

#### Mortality

Number of deaths recorded in each municipality for each year, gender and age group. Seven age groups were considered, as follows: under 1 year, 1-14, 15-44, 45-64, 65-74, 75-84 and 85 or over.

#### Population

Number of inhabitants living in each municipality by gender and age groups for each year.

#### Spanish mortality rate

Number of deaths per 10,000 inhabitants in Spain for each year, gender and age group. For children under 1 year of age, the mortality rate was recorded per 1,000 inhabitants.

### Sources of information

Mortality was derived from the Andalusian Deaths Registry. The number of inhabitants living in each municipality for each year between 1981 and 2002 was taken from the population estimates provided by the Andalusian Statistics Institute (IEA). From 2003 onwards, the resident population was taken from the Local Census.

The specific mortality rates in Spain for each gender, age group and year, as well as the Spanish population estimates between 1981 and 2002 were taken from National Statistics Institute (INE) data. From 2003 onwards, the number of inhabitants in Spain was taken from the Local Census.

### Statistical data analysis

Two spatio-temporal hierarchical Bayesian models were fitted for each gender and age group. One of these was used to estimate the specific mortality rate, together with its time trends, and the other to estimate the specific rate ratio of each municipality compared with Spain as a whole (Table 1). Both models have different *offsets*. As a result, the second model is necessary to obtain appropriate rate ratio posterior distributions given that, in general, Bayesian smoothing of specific rate ratio is different to the ratio of smoothed specific rates [11].

**Table 1:** Specification and parameterization of models for the spatio-temporal distribution of mortality from all causes in small-areas of Andalusia (Southern Spain)

	Specific mortalily rate	Specific mortality rate ratio
**Distribution**	*O*_*imt *_~ *Poisson*(*μ*_*imt*_)	*O*_*imt *_~ *Poisson*(*μ*_*imt*_)
**Mean**	*μ*_*imt *_= *p*_*imt *_*r*_*imt*_	*μ*_*imt *_= (*p*_*imt*_*R*_*it*_)*RR*_*imt*_
**Model**		
**Constant terms**	*β*_0*i *_~ *flat*, *β*_1*i *_~ *N*(0, 0.00001), *β*_2*i *_~ *N*(0, 0.00001)	
**Temporal random structure**		
*Structured effect*		
*Hyperparameter*		
**Spatial random structure**		
*Structured effect*		
*Non-structured effect*		
*Hyperparameters*		

Notation

*O*_*imt *_: deaths within age-gender subgroup *i *in municipality *m *at time *t*

*p*_*imt *_: population within age-gender subgroup *i *in municipality *m *at time *t*

*r*_*imt *_: specific mortality rate in municipality *m *at time *t*

*R*_*it *_: specific mortality rate in Spain at time *t*

: Median year

*RR*_*imt *_: specific rate ratio of town *m *compared with Spain as a whole at time *t*

*ω*_*mn*_, *m *≠ *n *: adjacency matrix (*ω*_*mn *_= 1 if *m *and *n *are neighbour areas and *ω*_*mn *_*= *0 otherwise)

In both models, it is assumed that the number of deaths observed within each age-gender subgroup *i*, municipality *m *and year *t *exhibit a Poisson distribution. The logarithm of the specific mortality rate and mortality rate ratio is expressed as the sum of a constant, a linear time effect, a quadratic time effect and two random spatial terms. One of these is unstructured and captures heterogeneity between municipalities, while the other is structured to account for the clustering of cases in space. Quadratic time effect captures most mortality trends [12], so it was included in Bayesian models. Possible cubic and upper time effects were tested using the DIC criterion [13].

Both models include a spatio-temporal parameterization which enables the trend of the specific mortality rate and the rate ratio for each municipality to be modelled [14]. This estimate also pinpoints those geographical municipalities that have experienced an increase or decrease in mortality rates over time for each age group and gender. Mathematical notation in Table 1 is usual in *Conditional Autoregressive Models *(CAR) [2,14,15]. Gamma, flat and Normal prior distributions were respectively used for precision parameters, intercept and model coefficients [14]. To avoid mathematical inconsistencies in mortality rates estimation, the value for the *mortality *variable was considered to be missing for all municipalities exhibiting both mortality and population equal to 0.

The model estimates were obtained by means of *Markov Chain Monte Carlo *(MCMC) algorithms, with 1000 iterations for burn-in and at least 10,000 later updates. The convergence was verified using two chains through the Gelman-Rubin statistical test as modified by Brooks and Gelman [16]. WinBUGS software was used for analysis [17].

To decide which municipalities have excess mortality, we applied a decision rule based on computing the probability that the specific rate ratio is greater than 1 with the following cut-off points: 0.05, 0.20, 0.80 and 0.95 [18].

Municipalities with values above 0.80 are small-areas at risk. A significant excess mortality was considered when probability was greater than 0.95. Probabilities between 0.2 and 0.8 show little evidence that the rate ratio is above 1, so the specific mortality rate of these municipalities is considered to be similar to the reference mortality rate. Municipalities with values below 0.20 are low-risk small-areas and municipalities with probability lower than 0.05 are considered small-areas with a significantly lower specific mortality rate than the Spanish national rate.

## Results

It was seen on the IEA data bases that there were some municipalities with a recorded number of deaths higher than the resident population for certain years between 1981 and 2006. For instance, for the age group under 1 year, there were five municipalities (0.6%) where male deaths outnumbered the resident male population and a further 13 municipalities (1.7%) showed the same pattern but for female deaths.

The same error was seen in 2 municipalities (0.3%) for male mortality in the 65-74 year -old age group, 10 municipalities (1.3%) for male mortality in the 75-84 year-old age group, 2 municipalities (0.3%) for female mortality in the 75-84 year-old age group, 149 municipalities (19.3%) for male mortality in the 85 years and over age group and a further 49 municipalities (6.4%) for female mortality in the 85 years and over group.

Given the high number of municipalities with this error in the 85 years and over age group, we decided that no analysis would be conducted on these data. For the remaining age groups, the value for the *mortality *variable was considered to be missing for all municipalities exhibiting this error.

### Trend for the specific mortality rate, 1981-2006

**Figure 1:**
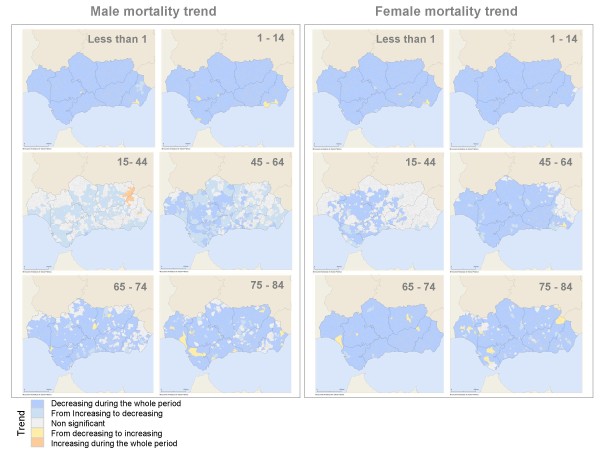
Mortality from all causes trend, 1981-2006.

During the study period, over 97% of municipalities exhibited a diminishing or stable mortality trend for all age and gender groups (Table 2). Around 2% of the municipalities showed a rising trend in both male and female mortality for the 75-84 year-old age groups. These municipalities are scattered on the map of Andalusia (Figure 1).

**Table 2:** Number and percentage of municipalities with decreasing, non-significant or increasing specific mortality trend during the period 1981-2006

	Specific mortality trend, 1981-2006		
Male age group	Decreasing	Non significant	Increasing
Under 1 year	767 (99.6%)	0 (0.0%)	3 (0.4%)
1-14	760 (98.7%)	1 (0.1%)	9 (1.2%)
15-44	243 (31.6%)	517 (67.1%)	10 (1.3%)
45-64	459 (59.6%)	311 (40.4%)	0 (0.0%)
65-74	546 (70.1%)	219 (28.4%)	5 (0.6%)
75-84	601 (78.0%)	150 (19.5%)	19 (2.5%)

	**Specific mortality trend, 1981-2006**		
**Female age group**	**Decreasing**	**Non significant**	**Increasing**

Under 1 year	766 (99.5%)	1 (0.1%)	3 (0.4%)
1-14	765 (99.4%)	4 (0.5%)	1 (0.1%)
15-44	171 (22.2%)	598 (77.7%)	1 (0.1%)
45-64	676 (87.8%)	93 (12.1%)	1 (0.1%)
65-74	761 (98.8%)	2 (0.3%)	7 (0.9%)
75-84	652 (84.7%)	102 (13.2%)	16 (2.1%)

### Geographical distribution of the specific mortality rate, 2006

**Figure 2:**
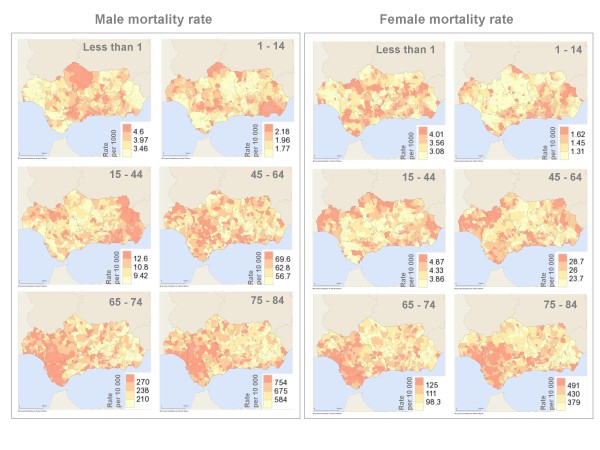
Mortality from all causes smoothed specific rate, 2006.

In 2006, the geographical distribution of the specific mortality rate was different for each age group (Figure 2). Each graph shows the division of the specific mortality rate into quartiles, using the darkest colour for the last quartile values.

For the age groups under 65 years of age, municipalities with higher mortality rates were seen to group geographically (Figure 2). However, the similarity between quartiles for each of the specific rates suggests a certain degree of homogeneity in these values for all Andalusian municipalities. Thus, the interquartile range (i.e. difference between the third and first quartiles) stands at between 0.3 deaths per 10,000 inhabitants for female mortality in the 1-14 year age group and at 12.9 per 10,000 inhabitants for male mortality in the 45-64 year age group (Figure 2).

For the 65-74 and 75-84 year-old age groups, municipalities in the Western half of Andalusia with the highest mortality rates in 2006 were also seen to group geographically (Figure 2). The interquartile range stood at between 26.7 and 170 deaths per 10,000 inhabitants which correspond respectively to female mortality for the 65-74 year-old age group and male mortality for the 75-84 year-old group.

### Trends in the specific mortality rate ratios, 1981-2006

**Figure 3:**
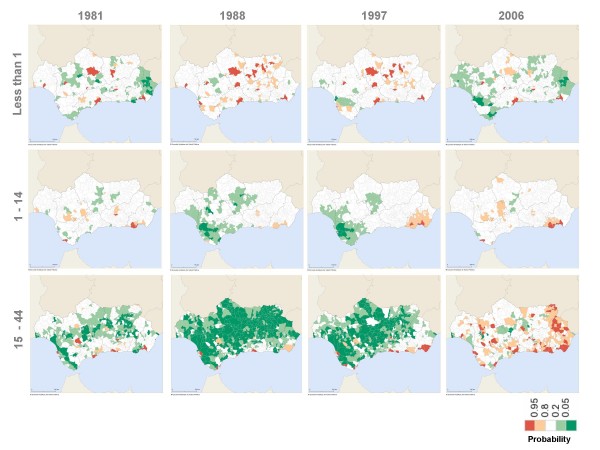
Probability that male mortality rate ratio is above 1 (under 45 age groups).

**Figure 4:**
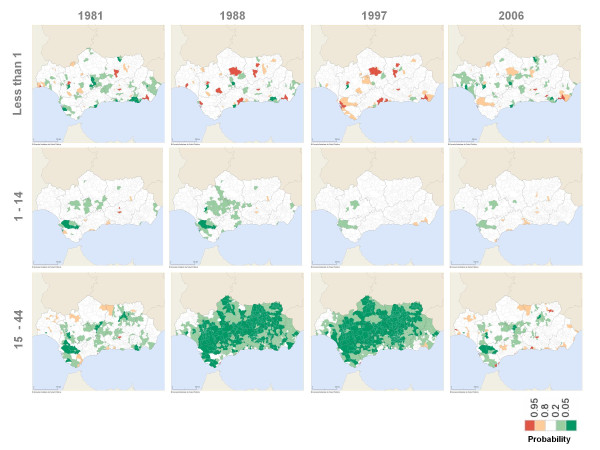
Probability that female mortality rate ratio is above 1 (under 45 age groups).

Between 1981 and 2006, the majority of Andalusian municipalities systematically had specific male and female mortality rates that were significantly lower or similar to those for Spain as a whole for the groups under 45 years of age (Figures 3 and 4).

**Figure 5:**
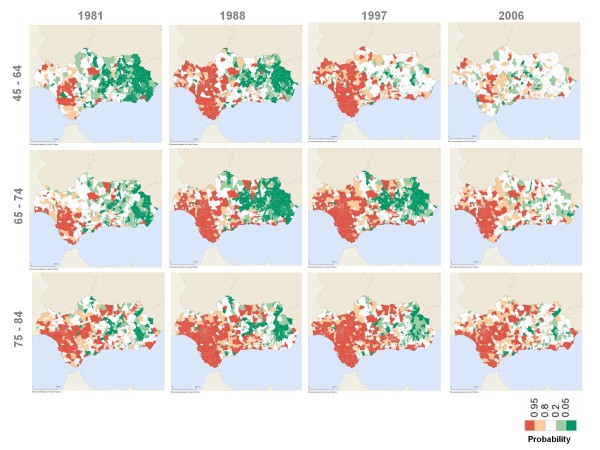
Probability that male mortality rate ratio is above 1 (over 45 age groups).

**Figure 6:**
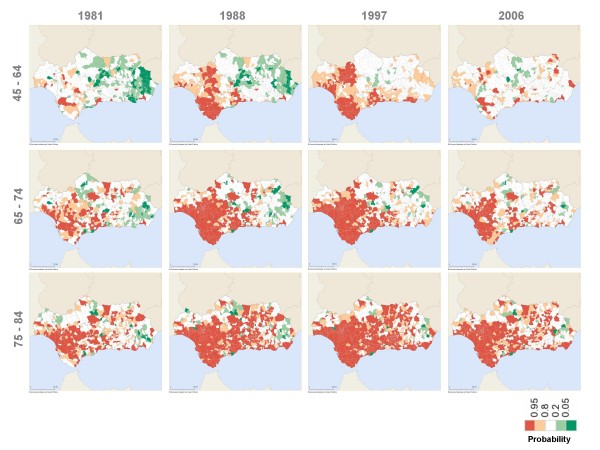
Probability that female mortality rate ratio is above 1 (over 45 age groups).

Between the second half of the 1980s and up to the end of the 90s, there was a group of Western Andalusian municipalities with mortality rates that were significantly higher than the national rates for Spain for the 45-64 year-old age groups for both genders. However, these significant differences gradually dissipated between 2000 and 2006 (Figures 5 and 6).

The analysis of the geographical distribution of the rates ratio for the 65-74 and 75-84 year-old age groups showed that, throughout the entire study period, those municipalities with rates significantly above the national rates for Spain were located in Western Andalusia, while municipalities with rates similar to or significantly lower than Spanish national rates were in Eastern Andalusia (Figures 5 and 6).

Although there are variations between gender and age groups, the increase in the number of municipalities with mortality rates that are similar to reference rates (in white), in general terms, show a trend towards convergence with the Spanish specific rates since the mid 1990s.

A summary of number of deaths per municipality showed high variability in all years and age groups (Table 3).

**Table 3:** Summary of mortality from all causes in Andalusia (Southern Spain)

	Male mortality																	
	<1 year-old age group						1-14 year-old age group						15-44 year-old age group					
	Municipalities		Deaths per municipality^1^				Municipalities		Deaths per municipality^1^				Municipalities		Deaths per municipality^1^			
Year	Valid^2^	Deaths	Min	Max	Mean	Sd	Valid^2^	Deaths	Min	Max	Mean	Sd	Valid^2^	Deaths	Min	Max	Mean	Sd
1981	646	959	0	113	1.48	7.37	768	457	0	36	0.60	2.66	770	2029	0	187	2.64	12.66
1988	741	509	0	32	0.69	2.62	770	289	0	23	0.38	1.59	770	2537	0	221	3.29	14.84
1997	734	261	0	31	0.35	1.77	770	135	0	9	0.18	0.67	770	2729	0	220	3.54	15.91
2006	710	237	0	33	0.33	1.65	770	112	0	9	0.15	0.59	770	2069	0	176	2.69	10.32
																		
	45-64 year-old age group						65-74 year-old age group						75-84 year-old age group					
	Municipalities		Deaths per municipality^1^				Municipalities		Deaths per municipality^1^				Municipalities		Deaths per municipality^1^			
Year	Valid^2^	Deaths	Min	Max	Mean	Sd	Valid^2^	Deaths	Min	Max	Mean	Sd	Valid^2^	Deaths	Min	Max	Mean	Sd
1981	770	7156	0	728	9.29	43.80	769	7838	0	713	10.19	42.27	757	8159	0	690	10.78	39.10
1988	770	7562	0	713	9.82	43.45	770	7756	0	690	10.07	42.94	770	9146	0	729	11.88	44.27
1997	770	6759	0	629	8.78	38.46	770	9277	0	814	12.05	49.73	770	9690	0	796	12.58	49.68
2006	770	5807	0	587	7.54	31.29	770	7416	0	666	9.63	36.10	770	11327	0	1007	14.71	54.80

	**Female mortality**																	

	<1 year-old age group						1-14 year-old age group						15-44 year-old age group					
	Municipalities		Deaths per municipality^1^				Municipalities		Deaths per municipality^1^				Municipalities		Deaths per municipality^1^			
Year	Valid^2^	Deaths	Min	Max	Mean	Sd	Valid^2^	Deaths	Min	Max	Mean	Sd	Valid^2^	Deaths	Min	Max	Mean	Sd
1981	644	671	0	63	1.04	4.48	768	298	0	23	0.39	1.59	769	815	0	67	1.06	4.70
1988	737	431	0	40	0.58	2.76	770	197	0	16	0.26	1.13	770	917	0	92	1.19	5.63
1997	721	206	0	21	0.29	1.38	770	142	0	10	0.18	0.73	770	1026	0	96	1.33	6.56
2006	702	169	0	18	0.24	1.12	767	74	0	4	0.10	0.38	770	860	0	98	1.12	4.81
																		
	45-64 year-old age group						65-74 year-old age group						75-84 year-old age group					
	Municipalities		Deaths per municipality^1^				Municipalities		Deaths per municipality^1^				Municipalities		Deaths per municipality^1^			
Year	Valid^2^	Deaths	Min	Max	Mean	Sd	Valid^2^	Deaths	Min	Max	Mean	Sd	Valid^2^	Deaths	Min	Max	Mean	Sd
1981	770	3593	0	389	4.67	22.48	770	5508	0	505	7.15	30.25	767	9732	0	877	12.69	50.97
1988	770	3398	0	324	4.41	20.38	770	5227	0	469	6.79	29.16	770	10646	0	954	13.83	55.70
1997	770	2882	0	280	3.74	17.14	770	5258	0	472	6.83	30.15	770	10573	0	982	13.73	57.56
2006	770	2481	0	276	3.22	14.77	770	3899	0	353	5.06	20.05	770	10589	0	986	13.75	55.31

1 Minimum (Min), Maximum (Max), Mean and Standard deviation (Sd)

2 Number of municipalities with population higher than 0 and with number of deaths equal or lesser than the resident population.

### Geographical distribution of the specific mortality rate ratio, 2006

In 2006, over 95% of municipalities showed male and female specific mortality rates that were similar or significantly lower than the Spanish national rates for the under 65 age groups.

Between 16% and 33% of municipalities showed significant excess mortality for the over 65 year-old age groups. In these municipalities, mainly grouped in Western Andalusia, the similarity between the first and third quartiles of the specific mortality rate and the rates ratio suggests a certain degree of homogeneity between municipalities in terms of mortality figures (Table 4).

**Table 4:** Number and characteristics of municipalities with excess mortality in 2006

	Male		Female	
	65-74	75-84	65-74	75-84
**Number of municipalities**	124 (16%)	172 (22%)	142 (18%)	251 (33%)
**Smoothed mortality rate per 10 000**				
Minimum	248.0	664.1	111.3	387.8
25	285.7	763.7	131.1	477.0
50	303.8	822.0	141.2	516.9
75	320.1	871.2	152.9	570.6
Maximum	488.8	1728.0	205.2	1646.0
**Smoothed rate ratio**				
Minimum	1.1	1.1	1.1	1.1
25	1.3	1.2	1.3	1.3
50	1.3	1.3	1.4	1.4
75	1.4	1.4	1.5	1.6
Maximum	2.2	2.8	2.1	4.6

**Spanish rate per 10 000**	221.2	595.6	96.8	352.8

## Discussion

This study independently analyses spatio-temporal trends of mortality in each of specific age by gender group in order to avoid well known problems related to standardized mortality rates and count data aggregated over time [3-7]. As a result, summary of mortality counts per year showed sparseness of the mortality outcome in some age-gender groups. However, it is precisely the small number of cases per municipality that makes it possible to model mortality through Poisson's distribution. Extra-Poisson variability or overdispersion, which could be caused by both structured and unstructured spatial heterogeneity, can be controlled by Generalised Linear Mixed Models, where Besag-York-Mollié Bayesian model is currently one of the most widely used [19-21]. This and other Bayesian methods, as used in this paper, enables smoothing rates when values of health outcome variable are small, having proven efficacy in a number of researches [22].

Results from this paper form part of the Interactive Mortality Atlas for Andalusia (AIMA) [23]. This is the first GIS to be devised in Spain that enables the geographical distribution and trends over time for the leading causes of death for both men and women to be viewed on a website. Data are examined and regularly updated, contributing towards broader understanding of the past and present of mortality in Andalusia from 1981. Health professionals have open access to AIMA in http://www.demap.es.

In this paper, maps for both specific mortality rate and specific mortality rate ratio show that the geographical distribution of mortality varies according to year, gender and age group. This finding is empirical proof of what has already been published in theoretical papers, namely, using lengthy periods grouping several years, standardised mortality ratio or age-adjusted mortality rates may not be appropriate to describe the geographical pattern of mortality [3,5-7].

Age-specific mortality analysis showed several geographical groupings of municipalities with higher (or lower) rates. However, small interquartile ranges in specific mortality rates of age groups under 65 years suggested little variation in geographical distribution for male and female mortality rates.

The interquartile range is a robust measure of the statistical dispersion of health indicators and should always be taken into account when interpreting rates or rates ratio maps. In other cases, choropleth maps might generate misinformation and undesirable scientific, political and social alarm. Unfortunately, most published papers tend to overlook this possibility.

Bearing this reflexion in mind, four items in the results should be stressed: Over 97% of the municipalities showed a diminishing or flat mortality trend in all gender and age groups. Mortality trends seem to converge with Spanish rates for a majority of municipalities. In 2006, over 95% of municipalities showed specific rates for male and female mortality that were similar or significantly lower than Spanish rates for all age groups fewer than 65. Systematically, municipalities in Western Andalusia showed significant male and female mortality excess from 1981 to 2006 only in the age groups over 65 years.

Due to the ecological design, it is not easy to account for these facts. Despite its popularity, this kind of analysis poses certain limitations that should be taken into account if the results obtained in any small-area epidemiology study are to be interpreted correctly.

Firstly, any statistical model allows the geographical distribution of mortality to be described, but it cannot explain the differences seen between municipalities. As all data is aggregate, we cannot know the level of exposure for any given risk factor for persons both dead and alive. Neither can we ascertain whether the individuals who currently reside in a given municipality have lived there most of their lives, and hence have been exposed to local environmental risk or prognosis factors. Therefore, any hypothesis suggesting a link between excess mortality detected in any of the municipalities and social inequities, use of healthcare services or environmental exposure, may lead to the ecological fallacy [24].

Secondly, in small-area epidemiology studies it is common to find an information bias linked to unregistered migrations that are not recorded on official information systems [25,26]. Studies conducted in Spain show that between 17% and 84% of deaths recorded in certain municipalities are for individuals who were not registered on the local census [27,28]. Further studies conducted in the U.S. revealed that 24% of deaths studied were recorded on the death certificate under an incorrect residence code [29]. Such flaws in information systems have led to major errors in estimating mortality rates. Similar facts in this paper led us to reject the analysis of mortality for the 85 years or over age group.

Such errors are not only found in mortality studies. Research conducted on hospital admissions, cancer incidence and other health indicators have also revealed flaws in information records. For instance, 24% of all hospital admissions at a hospital in southern Spain were patients who lived in the municipality but who were not officially registered as residents, leading to an overestimation of the hospital admission rate [30]. Likewise, certain studies have warned of major differences in figures for cancer incidence according to the denominator used in the calculation [31,32]. Research in the U.S. also shows that rates based on population estimates differ by some 60% from rates based on census data, which may lead to a difference in calculations for breast cancer incidence of up to 22% [32].

Since 1975, deaths in Spain have been classified according to place of residence and not place of death. No study has been conducted to date in Andalusia to assess the quality of data regarding municipality of residence and cause of death recorded on the Death Statistics Form. As a result, we should be extremely careful when interpreting mortality maps, or undertaking studies to correlate geographical distribution with different indicators or when positing hypotheses on the causes of death involved in differences in mortality between municipalities. Occasionally, inequalities in health and mortality recorded in small-area studies may only be the result of unregistered migratory flows that are not recorded in official population figures [33].

Taken into account these comments and the results from this paper, we have to be very cautious before suggesting an epidemiological cluster in Southern Spain concerning mortality in the age groups over 65 year. Excess mortality may only be the result of unregistered migratory flows [33] or, perhaps, it is the consequence of different lifestyles, health outcomes, socioeconomic status or contextual factors compared to people that live in municipalities with low mortality, as it has been described in other European countries [34-36]. Given that death is the endpoint of a past health record, mortality indicators should be complemented by other sources of information and individual-based studies that would provide an overall dynamic view of the population's health status [36].

Over the past few decades, advanced societies have undergone far-reaching changes linked to phenomena such as globalisation, influx of immigrant populations or aging of the population that have brought about major transformations in the demographic, economic and social structure of the different countries [37,38]. The magnitude of these changes has not always met with an organised response from Epidemiology Surveillance Systems. As a result, the updating, monitoring and analysis of health indicators poses a key challenge for Public Health, for the prevention and for control of the main health problems today, which are mostly linked to the new epidemics associated with social inequities or lifestyles seen in developed societies [39].

Dynamic mortality maps are able to provide up-to-date understanding of the geographical distribution and evolution over time of mortality, substantially improving on the information provided by conventional static atlases. However, a systematic validation of the information sources, a careful statistical analysis and a thorough interpretation of mortality maps are all still needed. By building this practice into future research, we will generate a useful procedure for health policy planning.

## Conclusions

More than 97% of the Andalusian municipalities showed a diminishing or flat mortality trend in all gender and age groups. Between 1981 and 2006, there was a dynamic geographical distribution of mortality, with a different pattern for each year, gender and age group. In 2006, over 95% of municipalities showed male and female mortality specific rates similar or significantly lower than Spanish rates for all age groups below 65. Systematically, municipalities in Western Andalusia showed significant male and female mortality excess from 1981 to 2006 only in age groups over 65.

Due to the ecological design, it is not easy to account for these facts. Taking into account the results from this paper and the previous comments in discussion section, we have to be very cautious before suggest an epidemiological cluster in South-western Spain concerning mortality in the age groups over 65 year. This study will contribute towards a reflection on the past, present and future of mortality in Andalusia.

## Competing interests

The authors declare that they have no competing interests.

## Authors' contributions

ROR conceived and coordinated the study, performed the WinBUGS code, participated in statistical analysis, and drafted the manuscript. JMMC participated in the design of the study, obtained data, and drafted the manuscript. All authors read and approved the final manuscript.

## Pre-publication history

The pre-publication history for this paper can be accessed here:

http://www.biomedcentral.com/1471-2458/10/26/prepub
